# Renin–Angiotensin–Aldosterone Axis Activity in Women with Chronic Endometritis: A Prospective Observational Study

**DOI:** 10.3390/ijms27188256

**Published:** 2026-09-16

**Authors:** Giulia Garbin, Loris Marin, Chiara Sabbadin, Giuseppe Gullo, Decio Armanini, Luciana Bordin, Eugenio Ragazzi, Guido Ambrosini, Alessandra Andrisani

**Affiliations:** 1 Department of Women’s and Children’s Health, University of Padova, 35128 Padova, Italy; giulia.garbin@studenti.unipd.it (G.G.); loris.marin@unipd.it (L.M.); guido.ambrosini@unipd.it (G.A.); alessandra.andrisani@unipd.it (A.A.); 2 Endocrine Unit, Department of Medicine DIMED, University of Padova, 35128 Padova, Italy; chiara.sabbadin@unipd.it; 3 Department of Obstetrics and Gynecology, Villa Sofia Cervello Hospital, University of Palermo, 90146 Palermo, Italy; gullogiuseppe@libero.it; 4 Studium Patavinum, University of Padova, 35122 Padova, Italy; eugenio.ragazzi@unipd.it

**Keywords:** chronic endometritis (CE), renin–angiotensin–aldosterone system (RAAS), aldosterone, mineralocorticoid receptor (MR), CD138, infertility

## Abstract

Chronic endometritis (CE) is a persistent inflammatory disorder of the endometrium that is histopathologically characterized by CD138-positive plasma cells within the endometrial stroma. This study evaluated components of the renin–angiotensin–aldosterone system (RAAS) and interleukin-6 (IL-6) in infertile women with CE. Thirty-nine infertile women undergoing diagnostic hysteroscopy with endometrial biopsy were included. A total of 21 women were classified as CE-positive and 18 as CE-negative. Fasting blood samples were collected 48 h before hysteroscopy (t1) and approximately 2.5 h before the procedure (t2) to assess plasma renin, serum aldosterone, the aldosterone-to-renin ratio (ARR), sodium, potassium, progesterone, thyroid-stimulating hormone (TSH), and IL-6. Exploratory analyses were performed according to the extent of CD138-positive plasma-cell infiltration (plasma-cell burden, stratified as <20 or ≥20 cells/high-power field) and persistence versus resolution of CE after antibiotic treatment. Baseline characteristics, including body mass index (BMI), infertility duration, and anti-Müllerian hormone (AMH), were comparable between groups. No significant differences in the parameters were observed between CE-positive and CE-negative women. No consistent RAAS or inflammatory differences emerged according to plasma-cell burden or CE persistence after treatment. These findings suggest that CE is not associated with measurable systemic RAAS activation or IL-6 changes, supporting the hypothesis of local CE alterations.

## 1. Introduction

Chronic endometritis (CE) is a persistent, subclinical inflammatory condition of the endometrium, histologically characterized by the presence of CD138/syndecan-1 positive plasma cells within the endometrial stroma [1,2]. Most affected women are asymptomatic, and the condition is frequently diagnosed during infertility evaluation, with recurrent implantation failure (RIF), recurrent pregnancy loss and impaired reproductive outcomes [3,4].

CE has been associated with a heterogeneous spectrum of microorganisms. In a large culture-based series of women with hysteroscopically suspected CE, at least one microorganism was recovered from 73.1% of endometrial samples [5]. Frequently detected organisms include *Escherichia coli*, *Enterococcus faecalis*, *Streptococcus* spp., *Staphylococcus* spp., *Mycoplasma* spp., and *Ureaplasma* spp., whereas *Chlamydia trachomatis* and *Neisseria gonorrhoeae* account for only a minority of cases [5,6,7,8]. Antibiotic treatment is commonly offered to women with histologically confirmed CE, although no universally accepted regimen exists. Empirical doxycycline is the most frequently used first-line treatment with a reported histological resolution rate of approximately 53% to 100% of cases [8]. Routine endometrial culture and culture-directed treatment are not standardized because of low microbial biomass, potential cervicovaginal contamination, and incomplete concordance with histology and molecular testing [6,7]. ESHRE considers CE assessment in women with RIF but does not establish culture as routine care, while the recent CE consensus suggests antibiogram testing in intractable or refractory cases [8]. However, microbial detection does not invariably establish causality, and CE may encompass both infectious and post-infectious inflammatory phenotypes [7]. Increasing evidence indicates that alterations in T-cell populations, abnormal plasma-cell recruitment, excessive cytokine production, and disruption of endometrial immune homeostasis play a key role in its pathogenesis [1,9,10]. Moreover, despite the local inflammatory nature of CE, the involvement of a low-grade systemic inflammation through the release of cytokines and immune mediators is still not proven [11]. Some studies have described altered circulating inflammatory profiles in women with recurrent implantation failure [12,13], whereas others have failed to detect significant differences [13,14].

The renin–angiotensin–aldosterone system (RAAS) has emerged as an important modulator of innate and adaptive immune responses [15,16]. We previously characterized mineralocorticoid receptors (MRs) in human circulating mononuclear leukocytes [17] and demonstrated that aldosterone exerts a pro-inflammatory effect in these cells, as evidenced by increased expression of inflammatory markers. Notably, this effect was abolished by coincubation with the MR antagonist canrenone [15], supporting the involvement of MR-mediated signaling. Aldosterone, acting through the mineralocorticoid receptor, promotes macrophage activation, T-cell polarization, and cytokine production, including interleukin-6 (IL-6), thereby linking endocrine regulation with immune activation [16]. MRs have been reported in cardiomyocytes, fibroblasts, endothelial cells, vascular smooth muscle cells and inflammatory cells [17,18]. Further studies have shown an involvement of aldosterone in gynecological conditions associated with inflammation and increased cardiometabolic risk, including the use of hormonal contraceptives, preeclampsia, uterine fibroids, and endometriosis [19,20], as well as polyendocrine metabolic ovarian syndrome (PMOS; formerly polycystic ovary syndrome, PCOS) [21]. The female reproductive tract itself contains renin, angiotensin, aldosterone and mineralocorticoid receptors. A direct effect of aldosterone has been identified in the ovary, endometrium, and uteroplacental tissues, regulating angiogenesis, decidualization, vascular remodeling, and tissue homeostasis through autocrine and paracrine mechanisms [22,23,24]. Experimental evidence has also demonstrated local aldosterone production by endometrial glands, suggesting that mineralocorticoid signaling may directly participate in endometrial physiology independently of the systemic RAAS [23]. CE represents an attractive model to explore whether persistent local inflammation is accompanied by endocrine activation involving the renin–angiotensin–aldosterone axis. The aim of the present study was to evaluate whether chronic endometritis is associated with systemic RAAS and circulating inflammatory changes in infertile women by assessing plasma renin, serum aldosterone, ARR, sodium, potassium and IL-6. We also investigated a possible association of systemic RAAS activation with the severity of endometrial plasma cell infiltration, quantified by the CD138-positive plasma cell count, and whether persistence of CE after antibiotic treatment identified a subgroup characterized by a distinct endocrine-inflammatory profile.

## 2. Results

### 2.1. Systemic RAAS and Inflammatory Profile According to CE Status

Thirty-nine women were included in the primary analysis: 18 were CE-negative and 21 were CE-positive. The groups were comparable with respect to BMI, age, duration of infertility and AMH. No between-group differences were detected in renin, aldosterone, ARR, sodium, potassium or IL-6 at either time point; the additional endocrine parameters were likewise comparable (Table 1).

Absolute effect-size estimates for renin, aldosterone, ARR and IL-6 ranged from 0.040 to 0.427. Thus, the primary analysis did not identify a reproducible systemic RAAS or inflammatory profile associated with CE.

### 2.2. Exploratory Analysis According to CD138-Positive Plasma-Cell Burden

The exploratory plasma-cell-burden analysis included 22 women, with 11 patients in each group. A higher BMI was observed in the group with ≥20 CD138-positive plasma cells in the unadjusted analysis, whereas the other clinical characteristics were comparable (Table 2). This BMI association did not remain statistically significant after correction for multiple comparisons.

The most notable RAAS finding was the higher mean aldosterone concentration at t2 in patients with ≥20 versus <20 CD138-positive plasma cells (395.0 ± 180.4 versus 253.2 ± 122.5 pmol/L; *p* = 0.055; Cohen’s *d* = 0.919). However, this borderline difference was absent at t1 (*p* = 0.934; Cohen’s *d* = 0.037) and was not accompanied by corresponding differences in renin or ARR. No coherent differences were observed in electrolytes or IL-6. Therefore, the isolated large effect estimate at t2 did not define a consistent systemic RAAS pattern according to the extent of plasma-cell infiltration.

### 2.3. Exploratory Analysis According to CE Outcome After Antibiotic Treatment

Among the 21 women diagnosed with CE, post-treatment histological follow-up was available for 17 (81.0%). CE was resolved in 9/17 women (52.9%): seven achieved histological resolution after first-line therapy and two additional women after second-line therapy. CE persisted in 8/17 women (47.1%). No statistically significant differences were detected in RAAS parameters, potassium, IL-6 or the additional endocrine parameters (Table 3). ARR at t2 was numerically higher in persistent CE and had a moderate-to-large standardized effect estimate (Cohen’s *d* = 0.732), but the values were highly dispersed and were not accompanied by concordant differences in renin or aldosterone.

Sodium was numerically higher in resolved CE than persistent CE at both time points, but the absolute differences were small: 141.0 ± 1.5 versus 139.3 ± 1.9 mmol/L at t1 (difference, 1.7 mmol/L; *p* = 0.075) and 140.6 ± 1.0 versus 139.6 ± 1.1 mmol/L at t2 (difference, 1.0 mmol/L; *p* = 0.097). Accordingly, the moderate-to-large standardized effect estimates for sodium (rank-biserial *r* = 0.554 at t1 and Cohen’s *d* = 0.926 at t2) did not correspond to clinically relevant absolute differences. Duration of infertility also showed a large effect estimate (Cohen’s *d* = 0.948; *p* = 0.074), but this exploratory estimate was based on only 17 women. Overall, CE persistence did not identify a distinct systemic RAAS or inflammatory phenotype.

### 2.4. Overall Effect-Size Pattern and Multiplicity-Adjusted Results

Across all three analyses, effect sizes for RAAS parameters and inflammatory markers were consistently small to negligible. In Table 1, Cohen’s *d* and rank-biserial correlation values for renin, aldosterone, ARR and IL-6 ranged from 0.040 to 0.427, around or below the threshold conventionally considered small. The borderline aldosterone finding in Table 2 showed a nominally large Cohen’s *d* of 0.919 at the second time point, but this was not accompanied by a corresponding effect at the first time point (Cohen’s *d* = 0.037) or by parallel differences in renin or ARR, suggesting sampling variability rather than a consistent biological signal. In Table 3, large effect sizes for sodium and duration of infertility should be interpreted cautiously, given the small group sizes (*n* = 9 and *n* = 8), where effect size estimates are known to be unstable and prone to upward bias; the absolute sodium difference between groups was less than 2 mmol/L at both time points, which is of no clinical relevance. Taken together, the effect size profile supports the absence of a meaningful systemic RAAS or inflammatory signal across all comparisons.

### 2.5. Multivariate Analysis of the Systemic Bio-Clinical Profile

**Figure 1:**
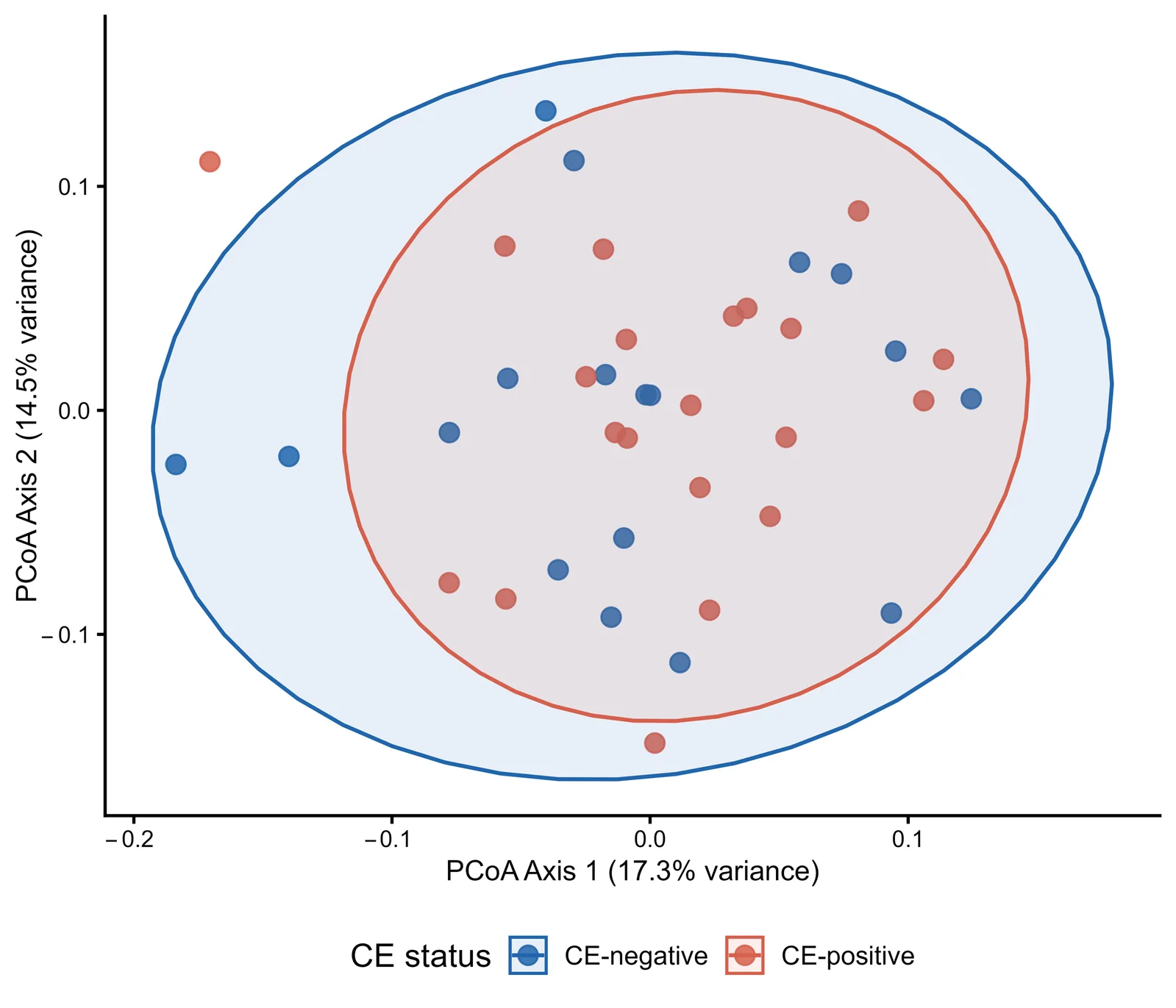
Principal coordinates analysis (PCoA) of systemic bio-clinical panel including RAAS and inflammatory parameters according to chronic endometritis status. Each point represents one patient. CE-negative patients are shown in blue; CE-positive patients in red. Ellipses represent 95% confidence regions estimated by *t*-distribution. Axis labels indicate the percentage of total variance explained by each principal coordinate. Analysis was performed on Gower distances computed across the available parameters measured at both time points. Missing values (6% of the total data matrix) were imputed using the missForest algorithm prior to distance computation. One CE-positive patient is a multivariate outlier, defined as a distance from the group centroid exceeding the mean plus two standard deviations. PERMANOVA: R^2^ = 0.017, F = 0.654, *p* = 0.763.

Principal coordinates analysis of the comprehensive systemic bio-clinical panel (encompassing circulating RAAS, inflammatory markers, electrolytes, BMI, and infertility duration) revealed substantial overlap between CE-positive and CE-negative patients in multivariate space (Figure 1). The first two axes accounted for 31.8% of the total variance (17.3% for Axis 1 and 14.5% for Axis 2). PERMANOVA confirmed the absence of statistically significant separation between groups (R^2^ = 0.017, F = 0.654, *p* = 0.763), indicating that CE status explained less than 2% of the multivariate variance in the overall bio-clinical profile. These findings support the univariate results, indicating that CE-associated endometrial inflammation does not correspond to a distinct systemic endocrine, inflammatory or clinical phenotype.

Notably, a single CE-positive patient emerged as a distinct multivariate outlier, lying well outside the 95% confidence ellipse of her diagnostic group along Axis 1. A review of her clinical profile revealed markedly elevated IL-6 concentrations at both time points (17.3 and 20.0 ng/L, respectively), which were approximately four to five standard deviations above the CE-positive group mean, in the absence of a correspondingly elevated RAAS profile. A subsequent review of her clinical history identified a transfusion-dependent hematological condition and atopic respiratory disease, both associated with chronic low-grade systemic inflammation, which had not been recognized as potential confounders at enrollment and were considered the most likely explanation for the extreme multivariate position. This patient was retained in the primary analysis, as her values were analytically verified and her exclusion would have introduced a post hoc selection bias.

## 3. Discussion

From our results, CE was not associated with significant alterations in circulating renin, aldosterone, ARR, electrolyte balance and IL-6 levels, showing that the inflammatory process underlying CE remains predominantly confined to the endometrial compartment. The lack of RAAS alterations was also found when evaluating CE-positive and CE-negative patients, plasma cell burden and persistence of CE after antibiotic treatment. Previous reports by our group and by others have also associated aldosterone and MR with inflammation, oxidative stress and immune modulation through mineralocorticoid receptor signaling [15,18,25,26]. The treatment of these clinical situations with aldosterone receptor antagonist can reverse or limit the cardiovascular and metabolic risk, even in the presence of normal aldosterone and renin. The persistence of CE after antibiotic treatment did not identify a subgroup with a clearly distinct systemic endocrine or inflammatory profile. The absence of systemic RAAS activation does not necessarily imply that mineralocorticoid signaling is irrelevant to CE pathophysiology and progression, but rather that their contribution occurs locally, as reported in other gynecological situations such as, for example, uterine fibroids, endometriosis and PMOS [19]. In some of these diseases associated with normal serum aldosterone concentrations, the treatment with spironolactone or other mineralocorticoid receptor antagonists is effective for decreasing inflammatory situations [27].

The endometrium is a highly specialized mucosal immune environment, sustaining tightly regulated local inflammatory responses while preserving systemic immune tolerance and reproductive function [28,29,30,31]. Several tissues express local renin–angiotensin pathways that operate through autocrine and paracrine mechanisms independently, at least in part, from the circulating RAAS [32]. Local steroid signaling and tissue RAAS activity may still contribute to inflammation, stromal remodeling and impaired receptivity. An important factor probably involved in the local action of aldosterone is the infiltration of inflammatory cells. These cells possess both glucocorticoid and mineralocorticoid receptors, involved in the response to the infection and related acute inflammation, leading to a resolution or to chronicization of the disease [33].

The association of normal aldosterone and of marker of systemic inflammation supports the absence of systemic biochemical signals associated with CE status.

We did not find alterations in the other general parameters evaluated as electrolytes, TSH, and progesterone, confirming our hypothesis that local gynecological inflammatory pattern is not associated with systemic inflammation. It is well known that the female gynecological apparatus is characterized by low immunological response allowing pregnancy. The lack of involvement of renin in this situation explains the lack of association with electrolyte dysfunction in tissues lacking 11β-hydroxysteroid dehydrogenase type 2 (11β-HSD2).

MR has similar affinity for aldosterone and cortisol, and in classical aldosterone-target tissues, ligand specificity is ensured by 11β-HSD2, which converts cortisol to cortisone and prevents inappropriate glucocorticoid-mediated MR activation [34,35]. An important role in the inflammatory pattern b CE is played by the invasion of circulating mononuclear cells bearing their MR and GR. In immune or stromal compartments, MR signaling may be influenced by local glucocorticoid availability rather than by circulating aldosterone concentrations [34]. We did not measure cortisol in our study, and we hypothesize that the normal ARR observed in our cohort can be consistent with local interplay of cortisol and aldosterone in situations of inflammation.

From these considerations, plasma cells and inflammatory cells infiltration remain largely restricted to the uterine microenvironment.

The borderline aldosterone difference at t2 in patients with higher plasma-cell burden (*p* = 0.055) was not accompanied by corresponding differences in renin or ARR and was absent at the first time point. Procedure-related anticipatory stress may contribute to explaining this discrepancy between the two sampling time points, as t2 was collected approximately 2.5 h before hysteroscopy and acute stress has been reported to transiently influence renin–aldosterone dynamics [36]. In addition, hematological parameters, including hemoglobin, hematocrit, and platelet count, were not assessed at t2 because they were not part of the standard fertility-care procedures. Therefore, there were possible variations in hydration status or hemoconcentration, as contributors to the biochemical measurements obtained at t2 could not be specifically evaluated.

This pattern is inconsistent with a sustained or reproducible aldosterone excess and most likely reflects sampling variability in a small exploratory subgroup.

Several limitations of this study should be acknowledged. First, no hematological parameters (hemoglobin, hematocrit, platelet count) were obtained at t2 in the present study. Hysteroscopy with endometrial biopsy is not hematologically neutral [36,37], and periprocedural hemoconcentration or volume shifts cannot therefore be excluded as potential contributors to the aldosterone values observed at t2, alongside the anticipatory stress-related mechanism discussed above. Second, no microbiological testing was performed to confirm the infectious component of CE; antibiotic treatment was administered empirically according to the institutional protocol, consistent with common clinical practice, but this precludes direct correlation between specific pathogens and the biochemical findings reported here. Third, systemic inflammatory assessment was mainly limited to IL-6 and did not include a broader cytokine panel.

An additional limitation is that no microbiological testing was performed to confirm the infectious component of CE; antibiotic treatment was administered empirically according to the institutional protocol, consistent with common clinical practice, but this precludes the direct correlation between specific pathogens and the biochemical findings reported here.

While the systemic inflammatory assessment was mainly limited to IL-6 and did not include a broader cytokine panel, the integrated endocrine approach adopted offers a solid framework for interpreting the absence of systemic RAAS activation in chronic endometritis. Future studies should therefore move toward direct characterization of endometrial RAAS components. Such an approach may clarify whether mineralocorticoid-related pathways contribute to CE through tissue-specific mechanisms that are not detectable in peripheral blood.

Furthermore, one CE-positive patient was identified post hoc as a distinct multivariate outlier on the basis of markedly elevated IL-6 concentrations. A review of her clinical records revealed comorbidities associated with chronic systemic inflammation that were not captured by the pre-specified exclusion criteria, highlighting the importance of prospective screening for occult inflammatory conditions in future studies of this type.

## 4. Materials and Methods

### 4.1. Study Design and Ethical Approval

This prospective, single-center observational clinical study was conducted at the Gynecology Unit of Padua University Hospital in collaboration with the Endocrinology Unit of the same institution, within the framework of the “Endometritis and Hyperaldosteronism” protocol (Ethics Committee Code: 5392/AO/22). The study was approved by the local Ethics Committee and conducted in accordance with the principles of the Declaration of Helsinki. All participants provided written informed consent before enrollment.

### 4.2. Participants and Eligibility Criteria

A total of 39 women with infertility undergoing diagnostic hysteroscopy as part of their infertility work-up were prospectively enrolled and followed between May 2023 and December 2025. Inclusion criteria were infertility, defined according to the World Health Organization (WHO) as failure to achieve pregnancy after at least 12 months of regular unprotected sexual intercourse [38], and age between 18 and 50 years.

The exclusion criteria were pregnancy, menopause, current estrogen–progestin therapy, ongoing hormonal stimulation, and systemic or local inflammatory conditions potentially affecting circulating inflammatory markers or RAAS activity.

Specifically, women were excluded in the presence of endometriosis, chronic inflammatory bowel disease, autoimmune disease, thyroid autoimmunity, including Hashimoto thyroiditis, acute infection or fever during the preceding four weeks, chronic kidney disease, liver disease, cardiovascular disease, arterial hypertension, or known adrenal disease.

Therefore, patients undergoing treatment with medications potentially interfering with RAAS assessment—including diuretics, mineralocorticoid receptor antagonists, glucocorticoids, angiotensin-converting enzyme inhibitors, angiotensin II receptor blockers, and beta-blockers—were also excluded. These criteria were adopted to minimize potential confounding effects on systemic inflammatory status, aldosterone secretion, renin concentrations, and electrolyte balance.

### 4.3. Diagnostic Hysteroscopy and Endometrial Sampling

All participants underwent diagnostic mini-hysteroscopy during the follicular or preovulatory phase of the menstrual cycle, preferably between cycle days 7 and 12.

Mini-hysteroscopy was performed using a lens-based mini-telescope with an outer diameter of 2.7 mm, equipped with a 3.5 mm outer-diameter single-flow diagnostic sheath (Karl Storz, Tuttlingen, Germany). During the procedure, an endometrial biopsy was obtained using a 3 mm Novak curette connected to a 20 mL syringe. The collected tissue was immediately placed in neutral buffered formalin for subsequent histological and immunohistochemical examination.

### 4.4. Histological and Immunohistochemical Assessment

Endometrial specimens were fixed in neutral buffered formalin and subsequently embedded in paraffin. Histological sections were stained with hematoxylin and eosin for conventional morphological evaluation. Additional 5 μm sections were separately processed for immunohistochemical staining using mouse anti-human monoclonal antibodies against CD138/syndecan-1 and multiple myeloma oncogene 1/interferon regulatory factor 4 (MUM1/IRF4). The monoclonal antibodies used were anti-CD138 clone MI15 (Cell Marque/Biocare Medical, Concord, CA, USA) and anti-MUM1 clone MRQ-8 (Cell Marque/Sigma-Aldrich, St. Louis, MO, USA). Immunostaining was performed according to validated routine protocols used by the Pathology Unit. CD138-positive cells located within the endometrial stroma were identified and counted as plasma cells. MUM1/IRF4 immunohistochemistry was additionally performed to confirm the plasma-cell nature of the inflammatory infiltrate [39]. All primary histopathological and immunohistochemical assessments were performed by a single pathologist.

According to the institutional diagnostic protocol and thresholds adopted in previous studies, CE was diagnosed when more than five CD138-positive endometrial stromal plasma cells per high-power field (HPF) were identified [40]. Five HPFs per specimen were evaluated by a single pathologist. For exploratory analyses, plasma-cell burden was defined as the extent of endometrial stromal plasma-cell infiltration based on the number of CD138-positive plasma cells per HPF.

### 4.5. Blood Sampling and Laboratory Measurements

Each participant underwent fasting venous blood sampling at two time points: 48 h before hysteroscopy and on the day of the procedure. The second blood sample (t2) was collected early in the morning, approximately 2.5 h before the hysteroscopic procedure.

Blood samples were obtained under standardized fasting conditions after a brief period of seated rest. Participants were instructed to maintain their usual sodium intake and to avoid sodium restriction before sampling.

Serum aldosterone, plasma direct renin, serum sodium, potassium, progesterone, thyroid-stimulating hormone (TSH), and IL-6 were measured at the Central Laboratory of Padua University Hospital according to validated laboratory procedures and the manufacturers’ instructions. Serum aldosterone and plasma direct renin were measured by automated chemiluminescent immunoassays on the LIAISON^®^ XL platform using the LIAISON^®^ XL Aldosterone and LIAISON^®^ Direct Renin assays, respectively (DiaSorin, Saluggia, Italy). Serum progesterone was measured by an electrochemiluminescence immunoassay (ECLIA) on the Modular DP platform (Roche Diagnostics, Mannheim, Germany). IL-6 was measured by an automated chemiluminescence immunoassay (CLIA) on the MAGLUMI 2000 Plus platform (Snibe Diagnostics, Shenzhen, China). The aldosterone-to-renin ratio (ARR) was calculated by dividing serum aldosterone by plasma direct renin and was used as an index of aldosterone secretion relative to its renin drive.

Sampling at two time points was intended to account for the short-term biological variability of aldosterone and renin related to posture, circadian rhythm, sodium intake, potassium status, and other physiological fluctuations. This design also allowed the reproducibility of any potential RAAS-related signal to be assessed rather than relying on a single isolated measurement [41,42].

### 4.6. Antibiotic Treatment and Histological Follow-Up

In the present study, antibiotic treatment and histological follow-up were provided as part of routine clinical care. First-line treatment consisted of oral doxycycline 100 mg twice daily for 14 days [8,43,44,45]. As part of the same routine-care pathway, a test-of-cure endometrial biopsy was performed no earlier than 10 days after completion of the antibiotic course. Follow-up sampling was scheduled within the same follicular-phase window as the diagnostic biopsy, preferably between cycle days 7 and 12. Histological resolution was defined as no longer meeting the institutional diagnostic threshold at follow-up, whereas persistent CE was defined as continued fulfillment of this criterion.

Women with persistent CE after first-line treatment received oral cefixime 400 mg once daily plus metronidazole 250 mg three times daily for 14 days. This locally adopted regimen was intended to extend antimicrobial coverage, particularly against Gram-negative and anaerobic microorganisms, after doxycycline failure. Because no universally accepted second-line regimen for CE currently exists, this therapeutic choice reflected institutional clinical practice [8].

### 4.7. Study Comparisons and Outcomes

The primary analysis compared CE-positive and CE-negative women according to the institutional diagnostic threshold of more than five CD138-positive stromal plasma cells/HPF. This analysis evaluated whether histologically diagnosed CE was associated with systemic RAAS activity, electrolyte balance, or circulating IL-6 concentrations.

A second exploratory analysis examined the overall burden of plasma-cell infiltration among women in whom more than one CD138-positive endometrial stromal plasma cell was identified, irrespective of whether they met the institutional diagnostic criterion for CE. Consequently, women were stratified into two groups according to plasma-cell burden: fewer than 20 versus ≥20 CD138-positive plasma cells/HPF. This analysis investigated whether the systemic endocrine, electrolyte, or inflammatory parameters varied according to the extent of the local plasma-cell infiltrate. The threshold of ≥20 cells/HPF was considered an exploratory marker of moderate-to-severe plasma-cell infiltration based on previously proposed semiquantitative classifications [46,47].

Finally, a third analysis was conducted among CE-positive women with available post-treatment histological follow-up. Women were classified as having resolved or persistent CE according to the institutional diagnostic threshold applied at repeat biopsy. This analysis evaluated whether histological persistence after antibiotic treatment was associated with a distinct systemic RAAS, endocrine, electrolyte, or inflammatory profile.

### 4.8. Statistical Analysis

Data were analyzed using R (R Core Team, 2026) and jamovi (The jamovi project, 2026) [48,49]. Continuous variables are presented as mean ± standard deviation (SD), including variables for which non-parametric tests were applied, to ensure consistency of presentation across tables. The normality of distribution was assessed for each variable using the Shapiro–Wilk test, applied separately within each comparison group. For normally distributed continuous variables, between-group comparisons were performed using Student’s *t*-test; when Levene’s test indicated significant heterogeneity of variances (*p* < 0.05), Welch’s *t*-test was applied instead, as it does not assume equality of variances. The Mann–Whitney U test was used for variables for which the normality assumption was not supported in at least one group. All statistical tests were two-sided, and a *p*-value < 0.05 was considered statistically significant. For each comparison, effect size was quantified using Cohen’s *d* for normally distributed variables and the rank-biserial correlation for non-parametric comparisons [50]. For Cohen’s *d*, effect sizes were interpreted according to conventional benchmarks—small (*d* = 0.2), medium (*d* = 0.5), and large (*d* = 0.8)—as proposed by Cohen [50]. For the rank-biserial correlation, the corresponding benchmarks were small (*r* = 0.1), medium (*r* = 0.3), and large (*r* = 0.5), as described by Cohen [50] and elaborated by Rosenthal [51]. Effect sizes are reported as absolute values.

No a priori sample size calculation was performed, as this study was designed as a prospective pilot investigation. A post hoc power analysis was conducted to characterize the minimum detectable effect size given the available sample, assuming α = 0.05 and 80% power. For the primary comparison between CE-positive and CE-negative patients, the study was adequately powered to detect only large effect sizes. For the exploratory subgroup analyses in Table 2 and Table 3, the minimum detectable effect size was higher, meaning that moderate or clinically meaningful differences may have gone undetected. These constraints should be considered when interpreting null findings, which cannot be equated with evidence of the absence of effect.

Given the large number of comparisons performed across the three analyses, approximately 57 in total, the expected number of false-positive results under the null hypothesis at α = 0.05 is approximately three. To address this, the Benjamini–Hochberg false-discovery rate procedure was applied across all comparisons. Under this correction, no parameter reached statistical significance. Given the declared exploratory and pilot nature of the study, uncorrected *p*-values are reported in the tables to preserve transparency; all findings should be interpreted as hypothesis-generating signals rather than as inferential conclusions.

To provide a multivariate, hypothesis-free characterization of the systemic biochemical profile, a principal coordinates analysis (PCoA) was performed on Gower distances computed across all circulating RAAS, electrolyte and inflammatory parameters, alongside key systemic clinical markers (BMI and duration of infertility) measured at both time points [52]. Gower distance was chosen to accommodate the mixed data nature and non-normal distributions of several continuous variables without requiring prior transformation. Missing values, representing 6% of the total data matrix, were imputed using the missForest algorithm prior to distance computation [53]. The statistical significance of group separation based on CE status was assessed by permutational multivariate analysis of variance (PERMANOVA) using the adonis2 function from the vegan package in R, with 999 permutations [54].

## 5. Conclusions

Chronic endometritis was not associated with measurable systemic activation of the renin–angiotensin–aldosterone system or with increased circulating IL-6 levels in infertile women undergoing diagnostic hysteroscopy. The lack of differences in renin, aldosterone and, most importantly, ARR indicates that systemic aldosterone function remains physiologically regulated despite the presence, severity or persistence of endometrial plasma cell infiltration. These findings support the concept of CE as a compartmentalized immune-inflammatory disorder, predominantly confined to the endometrial microenvironment. If mineralocorticoid-related pathways contribute to CE pathophysiology, they are more likely to operate through local endometrial mechanisms or tissue-specific RAAS signaling than through systemic endocrine activation.

## Figures and Tables

**Table 1:** Clinical characteristics, RAAS parameters and inflammatory markers according to chronic endometritis status.

Variable	CE-Negative (*n* = 18)	CE-Positive (*n* = 21)	*p*-Value ^a^	Effect Size ^b^
Clinical characteristics				
BMI (kg/m^2^)	24.92 ± 4.54	23.15 ± 2.69	0.225 ^†^	0.233
Age (years)	39.22 ± 4.21	38.76 ± 6.28	0.787 ^‡^	0.086
Duration of infertility (months)	51.39 ± 34.13	52.48 ± 37.17	0.931 ^†^	0.019
AMH (ng/mL)	1.93 ± 1.87	2.29 ± 2.30	0.759 ^†^	0.066
RAAS parameters				
Renin, t1 (mIU/L)	14.38 ± 8.60	16.36 ± 8.71	0.488	0.229
Renin, t2 (mIU/L)	17.74 ± 9.30	18.51 ± 13.03	0.839	0.067
Aldosterone, t1 (pmol/L)	276.0 ± 99.2	335.8 ± 171.5	0.188 ^‡^	0.427
Aldosterone, t2 (pmol/L)	374.1 ± 206.4	336.1 ± 160.7	0.534	0.207
ARR, t1	33.11 ± 31.69	31.49 ± 24.59	0.545 ^†^	0.133
ARR, t2	25.44 ± 14.40	34.83 ± 39.22	0.870 ^†^	0.040
Electrolytes				
Sodium, t1 (mmol/L)	140.0 ± 1.5	140.2 ± 1.8	0.546 ^†^	0.117
Sodium, t2 (mmol/L)	139.8 ± 2.1	140.2 ± 1.8	0.421 ^†^	0.153
Potassium, t1 (mmol/L)	4.00 ± 0.42	3.97 ± 0.29	0.790	0.089
Potassium, t2 (mmol/L)	4.04 ± 0.29	4.20 ± 0.45	0.204	0.421
Additional endocrine parameters				
TSH, t1 (mIU/L)	2.14 ± 0.82	1.81 ± 0.75	0.206	0.424
TSH, t2 (mIU/L)	2.23 ± 1.06	1.80 ± 0.75	0.156	0.470
Progesterone, t1 (nmol/L)	1.02 ± 1.05	0.85 ± 0.51	0.747 ^†^	0.064
Progesterone, t2 (nmol/L)	2.34 ± 5.05	2.23 ± 4.50	0.153 ^†^	0.278
Inflammatory marker				
IL-6, t1 (ng/L)	3.64 ± 0.93	4.40 ± 3.14	0.486 ^†^	0.134
IL-6, t2 (ng/L)	4.06 ± 1.84	4.56 ± 3.91	0.634 ^†^	0.094

Data are presented as mean ± SD. ^a^ Between-group comparisons were performed using Student’s *t*-test for normally distributed variables, with Welch’s correction applied when Levene’s test indicated heterogeneity of variances (‡), and the Mann–Whitney U test for non-normally distributed variables (†), as determined by the Shapiro–Wilk test applied separately within each comparison group. All *p*-values are two-sided, and *p* < 0.05 was considered statistically significant. ^b^ Effect sizes are reported as absolute values and are expressed as Cohen’s *d* for normally distributed variables, calculated using the pooled standard deviation under Welch’s correction where applicable, or rank-biserial correlation for non-parametric comparisons. Abbreviations: AMH, anti-Müllerian hormone; ARR, aldosterone-to-renin ratio; BMI, body mass index; CE, chronic endometritis; IL-6, interleukin-6; RAAS, renin–angiotensin–aldosterone system; SD, standard deviation; TSH, thyroid-stimulating hormone.

**Table 2:** Clinical and biochemical parameters according to CD138-positive endometrial plasma-cell burden.

Variable	<20 CD138-Positive Plasma Cells (*n* = 11)	≥20 CD138-Positive Plasma Cells (*n* = 11)	*p*-Value ^a^	Effect Size ^b^
Clinical and histological characteristics				
CD138-positive plasma cells, mean number	8.36 ± 3.83	25.45 ± 12.14	--	--
BMI (kg/m^2^)	22.20 ± 2.40	24.60 ± 2.40	0.034 ^†^	0.555
Age (years)	37.80 ± 6.60	39.80 ± 5.90	0.462	0.320
Duration of infertility (months)	54.55 ± 46.59	53.64 ± 28.55	0.417 ^†^	0.207
AMH (ng/mL)	1.54 ± 1.36	2.69 ± 2.79	0.689 ^†^	0.125
*RAAS parameters*				
Renin, t1 (mIU/L)	17.77 ± 10.10	13.64 ± 8.39	0.319	0.448
Renin, t2 (mIU/L)	16.11 ± 12.63	19.08 ± 14.38	0.630	0.219
Aldosterone, t1 (pmol/L)	344.2 ± 139.2	337.8 ± 198.1	0.934	0.037
Aldosterone, t2 (pmol/L)	253.2 ± 122.5	395.0 ± 180.4	0.055	0.919
ARR, t1	33.48 ± 40.38	33.92 ± 29.35	0.468 ^†^	0.200
ARR, t2	33.31 ± 43.35	32.32 ± 25.91	0.579 ^†^	0.160
*Electrolytes*				
Sodium, t1 (mmol/L)	140.1 ± 1.6	140.2 ± 2.1	0.668 ^†^	0.121
Sodium, t2 (mmol/L)	140.6 ± 2.2	139.7 ± 1.4	0.533 ^†^	0.172
Potassium, t1 (mmol/L)	4.14 ± 0.37	3.88 ± 0.25	0.084	0.798
Potassium, t2 (mmol/L)	4.29 ± 0.49	4.17 ± 0.41	0.570	0.260
Additional endocrine parameters				
TSH, t1 (mIU/L)	1.75 ± 1.01	1.99 ± 0.62	0.540	0.273
TSH, t2 (mIU/L)	1.54 ± 0.93	1.95 ± 0.62	0.251	0.533
Progesterone, t1 (nmol/L)	0.96 ± 0.41	0.80 ± 0.63	0.488	0.309
Progesterone, t2 (nmol/L)	1.63 ± 2.09	2.98 ± 6.17	0.910 ^†^	0.040
Inflammatory marker				
IL-6, t1 (ng/L)	4.89 ± 4.50	3.96 ± 1.00	0.695 ^†^	0.109
IL-6, t2 (ng/L)	4.82 ± 5.35	4.16 ± 1.42	0.354 ^†^	0.250

Data are presented as mean ± SD. ^a^ Between-group comparisons were performed using Student’s *t*-test for normally distributed variables and the Mann–Whitney U test for non-normally distributed variables (†), as determined by the Shapiro–Wilk test applied separately within each comparison group. All *p*-values are two-sided, and *p* < 0.05 was considered statistically significant. ^b^ Effect sizes are reported as absolute values and are expressed as Cohen’s *d* for normally distributed variables or rank-biserial correlation for non-parametric comparisons. Abbreviations: AMH, anti-Müllerian hormone; ARR, aldosterone-to-renin ratio; BMI, body mass index; CE, chronic endometritis; IL-6, interleukin-6; RAAS, renin–angiotensin–aldosterone system; SD, standard deviation; TSH, thyroid-stimulating hormone. -- = not applicable, as CD138-positive plasma cell count was the a priori criterion used to define the comparison groups.

**Table 3:** Clinical characteristics, RAAS parameters and inflammatory markers according to chronic endometritis outcome after antibiotic treatment.

Variable	CE Resolved (*n* = 9)	CE Persistent (*n* = 8)	*p*-Value ^a^	Effect Size ^b^
Clinical and histological characteristics				
CD138-positive plasma cells, mean number	13.44 ± 6.33	18.00 ± 7.25	0.285 ^†^	0.306
BMI (kg/m^2^)	22.20 ± 3.00	24.10 ± 1.90	0.112 ^†^	0.472
Age (years)	36.00 ± 5.90	39.80 ± 5.80	0.209	0.638
Duration of infertility (months)	61.33 ± 33.65	37.75 ± 10.33	0.074 ^‡^	0.948
AMH (ng/mL)	1.90 ± 1.89	2.18 ± 2.63	0.816	0.125
RAAS parameters				
Renin, t1 (mIU/L)	15.20 ± 9.91	17.76 ± 9.32	0.602	0.266
Renin, t2 (mIU/L)	16.00 ± 9.32	14.20 ± 11.75	0.750	0.168
Aldosterone, t1 (pmol/L)	339.6 ± 136.2	355.4 ± 239.9	0.874	0.081
Aldosterone, t2 (pmol/L)	295.5 ± 221.8	354.0 ± 147.2	0.552	0.316
ARR, t1	35.51 ± 33.66	22.37 ± 12.72	0.574 ^†^	0.188
ARR, t2	23.99 ± 16.79	50.69 ± 48.75	0.181 ^‡^	0.732
Electrolytes				
Sodium, t1 (mmol/L)	141.0 ± 1.5	139.3 ± 1.9	0.075 ^†^	0.554
Sodium, t2 (mmol/L)	140.6 ± 1.0	139.6 ± 1.1	0.097	0.926
Potassium, t1 (mmol/L)	4.10 ± 0.46	3.96 ± 0.30	0.491	0.353
Potassium, t2 (mmol/L)	4.26 ± 0.34	4.06 ± 0.36	0.303	0.556
Additional endocrine parameters				
TSH, t1 (mIU/L)	1.96 ± 1.06	1.86 ± 0.82	0.834	0.107
TSH, t2 (mIU/L)	1.81 ± 0.83	1.94 ± 0.88	0.775	0.151
Progesterone, t1 (nmol/L)	1.01 ± 0.67	0.71 ± 0.42	0.382 ^†^	0.281
Progesterone, t2 (nmol/L)	3.68 ± 7.42	1.01 ± 0.48	1.000 ^†^	0.018
Inflammatory marker				
IL-6, t1 (ng/L)	4.04 ± 1.45	5.50 ± 4.82	0.832 ^†^	0.078
IL-6, t2 (ng/L)	3.93 ± 1.58	5.96 ± 5.73	0.376 ^†^	0.286

Data are presented as mean ± SD. ^a^ Between-group comparisons were performed using Student’s *t*-test for normally distributed variables, with Welch’s correction applied when Levene’s test indicated heterogeneity of variances (‡), and the Mann–Whitney U test for non-normally distributed variables (†), as determined by the Shapiro–Wilk test applied separately within each comparison group. All *p*-values are two-sided, and *p* < 0.05 was considered statistically significant. ^b^ Effect sizes are reported as absolute values and are expressed as Cohen’s *d* for normally distributed variables, calculated using the pooled standard deviation under Welch’s correction where applicable, or rank-biserial correlation for non-parametric comparisons. Abbreviations: AMH, anti-Müllerian hormone; ARR, aldosterone-to-renin ratio; BMI, body mass index; CE, chronic endometritis; IL-6, interleukin-6; RAAS, renin–angiotensin–aldosterone system; SD, standard deviation; TSH, thyroid-stimulating hormone. CE outcome was assessed by repeat endometrial biopsy after one or two courses of antibiotic treatment.

## Data Availability

The original contributions presented in this study are included in the article. Further inquiries can be directed to the corresponding author.

## Abbreviations

The following abbreviations are used in this manuscript:

CE	Chronic endometritis
IL-6	Interleukin-6
MR	Mineralocorticoid receptor
RAAS	Renin–angiotensin–aldosterone system
ARR	Aldosterone-to-renin ratio
AMH	Anti-Müllerian hormone
TSH	Thyroid-stimulating hormone
BMI	Body mass index
